# Low-Dose Aspirin for Primary Prevention of Cardiovascular Events Comparing East Asians With Westerners: A Meta-Analysis

**DOI:** 10.1016/j.jacasi.2023.07.008

**Published:** 2023-09-12

**Authors:** Rock Bum Kim, Ang Li, Ki-Soo Park, Yune-Sik Kang, Jang-Rak Kim, Eliano P. Navarese, Diana A. Gorog, Udaya S. Tantry, Paul A. Gurbel, Jin Yong Hwang, Oh-Young Kwon, Young-Hoon Jeong

**Affiliations:** a Regional Cardiocerebrovascular Disease Center, Gyeongsang National University, Jinju, South Korea; b Section of Hematology-Oncology, Baylor College of Medicine, Houston, Texas, USA; c Department of Preventive Medicine and Institute of Health Sciences, Gyeongsang National University College of Medicine, Jinju, South Korea; d Interventional Cardiology and Cardiovascular Medicine Research, Department of Cardiology and Internal Medicine, Nicolaus Copernicus University, Bydgoszcz, Poland; e Postgraduate Medical School, University of Hertfordshire, Hertfordshire, United Kingdom; f National Heart and Lung Institute, Imperial College, London, United Kingdom; g Sinai Center for Thrombosis Research and Drug Development, Sinai Hospital of Baltimore, Baltimore, Maryland, USA; h Division of Cardiology, Department of Internal Medicine, Gyeongsang National University Hospital, Jinju, South Korea; i Department of Neurology, Gyeongsang National University Hospital, Jinju, South Korea; j CAU Thrombosis and Biomarker Center, Heart and Brain Hospital, Chung-Ang University, Gwangmyeong Hospital, Gwangmyeong, South Korea; k Department of Internal Medicine, Chung-Ang University School of Medicine, Seoul, South Korea

**Keywords:** aspirin, cardiovascular disease, East Asia, primary prevention

## Abstract

**Background:**

East Asians have shown different risk profiles for both thrombophilia and bleeding than Western counterparts.

**Objectives:**

The authors sought to evaluate the effect of low-dose aspirin for primary prevention between these populations.

**Methods:**

We searched randomized clinical trials (RCTs) for intervention with low-dose aspirin (≤100 mg once daily) in participants without symptomatic cardiovascular disease until December 31, 2021. The number of events between the arms was extracted for analysis. Pooled risk ratios (RRs) and risk differences (RDs) were analyzed in each population. Outcomes included a major adverse cardiovascular event (MACE), cardiovascular death, myocardial infarction, stroke, and major bleeding (intracranial hemorrhage and major gastrointestinal bleeding).

**Results:**

Two RCTs included 17,003 East Asians, and 9 RCTs had 117,467 Western participants. Aspirin treatment showed a similar effect in reducing the MACE rate (RR of East Asians: 0.87; 95% CI: 0.71-1.05; RR of Westerners: 0.90; 95% CI: 0.85-0.95) (*P*_interaction_ = 0.721). In contrast, the risk of major bleeding during aspirin vs control was greater in the East Asian population (RR: 2.48; 95% CI: 1.86-3.30) compared with the Western population (RR: 1.45; 95% CI: 1.26-1.66) (*P*_interaction_ = 0.001), which was driven by more frequent gastrointestinal bleeding (RR of East Asians: 3.29; 95% CI: 2.26-4.80 vs RR of Westerners: 1.56; 95% CI: 1.29-1.88) (*P*_interaction_ < 0.001). The net RDs (RD of MACE plus RD of major bleeding) were 8.04 and 0.72 per 1,000 persons in East Asian and Western participants, indicating 124 and 1,389 of the net number needed to harm, respectively.

**Conclusions:**

Low-dose aspirin for primary prevention in East Asians must be cautiously prescribed because of the increased risk of major bleeding relative to Western counterparts.

Although aspirin was known to have a preventive effect on the first occurrence of cardiovascular disease (CVD) until the early 2000s,^1^ this effect has become weaker in recent years. More recently, several meta-analyses, including the recent randomized clinical trials (RCTs), have reported an increased risk of bleeding from aspirin than potential benefits for primary prevention.^2^, ^3^, ^4^ Therefore, the primary preventive effect of aspirin remains uncertain even in high-risk cohorts. As clinical evidence for aspirin use accumulates over time, the strength of recommendation for primary prevention with aspirin has also changed. In 2009, the U.S. Preventive Services Task Force (USPSTF) recommended aspirin for the prevention of CVD as an A grade drug for men aged 45 to 79 years and women aged 55 to 79 years.^5^ In 2016, the USPSTF recommended aspirin as a B grade drug for a population aged 50 to 59 years.^6^ The 2022 USPSTF recommendation suggested its role for primary prevention as a C grade drug for adults aged 40 to 59 years.^7^

Multiple lines of epidemiologic evidence have suggested that the prevalence of CVD and its related mortality differ across ethnicities.^8^ The medical therapy recommended from the American and European guidelines has been developed based on clinical evidence collected from the Western population. The unique incidence and mortality of CVD in East Asians can be important to determine the optimal medical strategy. Compared with the Western population, the East Asian population has a different profile of cardiovascular (CV) risk factors, a lower incidence of coronary artery disease (CAD), and a higher rate of hemorrhagic stroke.^9^, ^10^, ^11^ Therefore, East Asian patients have shown a limited benefit in reducing ischemic events and an increased tendency of bleeding during antithrombotic therapy than Western subjects.^8^^,^^12^^,^^13^ Furthermore, East Asians generally have shown different responses to antithrombotic regimens compared with Caucasians.^13^^,^^14^ These findings of the “East Asian Paradox” can explain the unique risk-benefit profile in the East Asian population and support the introduction of tailored precision medicine during antithrombotic treatment.^12^, ^13^, ^14^ For this reason, its efficacy and safety in patients without established CVD need to be evaluated across these ethnicities. Therefore, we aimed to measure the benefits and harmful effects of low-dose aspirin (≤100 mg once daily) in East Asian and Western populations.

## Methods

This study was conducted according to the Preferred Reporting Items for Systematic Reviews and Meta-Analyses guidelines.^15^ This study was registered in PROSPERO (CRD42022309702).

### Data sources and searches

We considered all RCTs evaluating low-dose aspirin as a primary intervention in participants without prior CV events. Those with chronic diseases such as hypertension, diabetes mellitus, and dyslipidemia were allowed. The main results included CV outcomes according to major adverse cardiovascular events (MACEs), CV death, myocardial infarction (MI), stroke, and bleeding events.

We searched articles to include in the study using 3 electronic databases (PubMed [MEDLINE], Embase, and the Cochrane Library) with publication dates up to December 31, 2021, and without language restrictions. The search terms based on the PICOT (patient, intervention, comparison, outcome, and time) strategy were as follows: (prevent∗ AND aspirin∗ AND placebo∗ AND cardiovascular diseases AND clinical trials). The detailed search terms in PubMed, Embase, and the Cochrane Library are presented in Supplemental Table 1. Additionally, we used the snowball method to search reference lists of previous meta-analyses and empirical papers to find references that did not appear in the electronic search.

### Study selection

**Figure 1:**
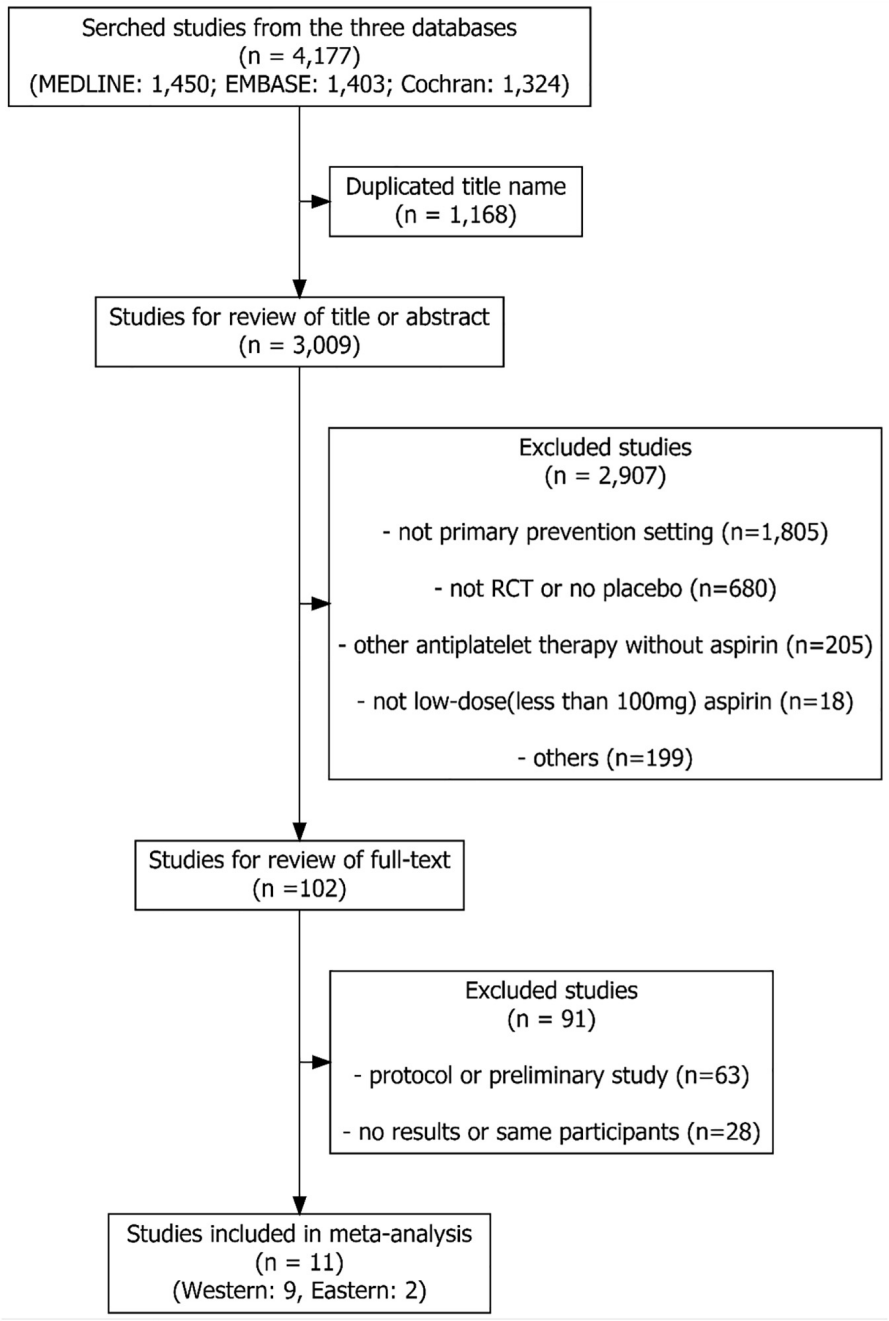
Flow of Study Selection This flow represents how we selected the acceptable studies that were analyzed in this meta-analysis. Using the key words, the first selected 4,177 studies were listed and deleted by Excel software (Microsoft) when their title was duplicated. After that, roughly 3,009 studies were reviewed by authors for checking the inclusion/exclusion criteria. Finally, 11 studies were selected after a review of full text of 102 studies. RCT = randomized clinical trial.

We selected eligible studies from the literature according to the following criteria: 1) the study participants had no CV events such as CAD, heart failure, or stroke; 2) the study design was a randomized placebo-controlled or nonplacebo trial; 3) the dose of aspirin was ≤100 mg once daily; 4) the control group consisted of placebo- or non–aspirin-treated participants; and 5) the outcome of the study was a CV event. The criteria for exclusion from this analysis were as follows: 1) an individual had pre-existing symptomatic CVD; 2) use of antithrombotic agents other than aspirin in the active arm; or 3) use of other antithrombotic agents in the control group. Two authors (R.B.K. and Y.-H.J.) independently conducted the literature search, screening of abstracts, and selection of the included trials. Potential disagreements in the review and selection of studies were discussed and resolved through consensus. A flowchart of the selection process is shown in Figure 1.

### Quality assessment and data extraction

Two reviewers independently assessed the selected studies using the Cochrane risk of bias tool.^16^ The tool is based on 7 items of bias assessment: random sequence generation, allocation concealment, blinding of participants, blinding of outcome assessment, incomplete outcome data, selective reporting, and other biases. The risk of bias in each study was classified as high risk, low risk, or unknown (insufficient information provided). There was a high-risk bias for blinding some studies. However, no study was excluded based on a high-risk bias.

We extracted data from each of the included studies and summarized the results. Data consisted of the characteristics, including ethnicity, of the participants and the main outcomes of each study. In addition, to assess the certainty of the evidence from the outcomes, we rated the quality of each outcome using the Grading of Recommendations, Assessment, Development and Evaluation approach.^17^ Grading was conducted using GRADEpro GDT (GRADE Working Group).

### Outcomes definition

The primary CV outcome was MACE, a composite of CV death, nonfatal MI, and nonfatal stroke.^7^ Secondary CV outcomes included CV death, MI, and total stroke (ischemic and hemorrhagic events if possible). The primary bleeding outcome was major bleeding, which was composed of intracranial hemorrhage (ICH) and major gastrointestinal (GI) bleeding. Secondary bleeding outcomes included each episode from ICH or major GI bleeding.

CV death was defined as death caused by heart and vascular diseases such as myocardial ischemia, heart failure, cardiac arrest, and stroke. MI events were defined as fatal or nonfatal without death. Stroke events were defined as ischemic or hemorrhagic, which were infarction or bleeding of the cerebral artery, respectively. ICH included all types of brain hemorrhages such as hemorrhagic stroke as well as epidural, subdural, or subarachnoid hematoma. Major GI bleeding was defined as bleeding of the upper or lower GI tract that led to hospitalization, transfusion, procedure, surgery, or death.

### Ethical approval

Institutional Review Board approval was not required because this systematic study was not conducted on people but instead on published literature.

### Statistical analysis

We calculated the pooled risk ratio (RR) and corresponding 95% CIs of the overall outcomes of the 2 ethnic groups regarding the benefits and harmful effects of aspirin. The meta-analysis was conducted using the fixed effects model or the random effects model. Heterogeneity was evaluated using Cochran’s Q test and the *I*^2^ statistic test. A *P* value of ≤0.10 on the Q test or an *I*^2^ value ≥50% was assumed to have heterogeneity. In this case, the random effects model was used to analyze the pooled effect size; otherwise, the fixed effects model was used.

To assess the difference in outcomes based on the participants’ ethnicities, we conducted subgroup analyses using the Borenstein method^18^ with a random effects model. We then calculated the pooled risk difference (RD) by calculating the difference between the proportion of outcome incidence in the aspirin and control arms. This RD was presented as per 1,000 persons instead of a percentage because of its low value. We also calculated the number needed to treat to benefit (NNTB) for MACEs or the number needed to treat harm (NNTH) for major bleeding using the inverse of the RD. For easy interpretation, we calculated the net RD by adding the RD of MACEs to the RD of major bleeding and the net number needed to treat by: 1/(1/NNTB − 1/NNTH).^19^

To assess the publication bias, we visually reviewed the funnel plot using the trim-and-fill method and calculated the potential publication bias–compensated effect size using Egger’s test. Furthermore, to confirm the effects of high-risk studies, we performed a sensitivity analysis by recalculating the pooled effect by eliminating each study one by one (leave-one-out method). The statistical programs used were Stata Statistical Software (StataCorp) and R software version 4.1.3 (R Foundation for Statistical Computing). A 2-tailed *P <* 0.05 was considered statistically significant.

## Results

### Study searching and characteristics

We found 4,177 human studies in the electronic databases according to our key words. After removing 1,168 duplicated studies, we reviewed 3,009 studies based on their titles or abstracts. Among them, we excluded 2,907 studies that did not match the inclusion criteria. The full texts of the remaining 102 studies were reviewed, and 91 were excluded because of the protocol or preliminary nature of the study or because there were no full-text articles. Finally, 11 studies^20^, ^21^, ^22^, ^23^, ^24^, ^25^, ^26^, ^27^, ^28^, ^29^, ^30^ were chosen for analysis (Figure 1).

The 11 studies included 134,470 participants, among which the aspirin group consisted of 67,157 participants and the placebo or nontreatment group consisted of 67,313 participants. The ethnicities of the participants were Westerners in 9 studies^20^, ^21^, ^22^, ^23^, ^24^, ^25^, ^26^, ^27^, ^28^ and East Asians in 2 studies.^29^^,^^30^ None of the participants had prior CVD, but the participants in some studies^20^, ^21^, ^22^^,^^24^^,^^26^, ^27^, ^28^, ^29^, ^30^ had risk factors such as hypertension, dyslipidemia, and diabetes mellitus (Table 1).

**Table 1:** Characteristics of Selected Studies to Analysis

Study (Ref. #)	Year	Country	Race or Ethnicity, %	Aspirin/Placebo				Aspirin Dose in the Active Arm	Study Population, %
				Number of Participants	Mean Age, y	Proportion of Male, %	Mean Follow-Up Period, y		
HOT^20^	1998	Multination (26 countries)	Europe/Israel (80.3) U.S./Canada (18.1) Mexico/Argentina (0.5) East Asia (1.1)	9,399/9,391	61.5	53.0	3.8	75 mg/d	HTN (100), DM (8.4)
TPT^21^	1998	United Kingdom	—	1,268/1,272	57.7/57.3	100.0/100.0	6.38/6.34	75 mg/d	HTN (18), DL (1)
PPP^22^	2001	Italy	Italian	2,226/2,269	64.5/64.3	43.0/42.0	3.6	100 mg/d	HTN (68), DL (39), DM (17)
WHS^23^	2005	United States	White (94.8) Hispanic (1.1) African American (2.3) Asian Pacific (1.4) American Indian (0.3) Other (0.2)	19,934/19,942	54.6/54.6	0.0/0.0	10.1	100 mg/alternate day	HTN (26.2), DL (27.2), DM (2.6)
POPADAD^24^	2008	Scotland	—	638/638	60.5/60.1	44.8/43.4	6.7	100 mg/d	DM (100)
AAA^25^	2010	Scotland	—	1,675/1,675 (ABI ≤ 0.95)	62.2/61.7	29.0/28.0	8.2	100 mg/d	HTN (37.8),^a^ lipid-lowering agents (4.2), DM (3)
ASCEND^26^	2018	United Kingdom	White (96.5) Indian/Pakistani/Bangladeshi (1.0) African, Caribbean (1.0) Other (1.0)	7,740/7,740	63.2/63.3	62.6/62.5	7.4	100 mg/d	HTN (61.6), statin use (75.3), type 2 DM (94.1)
ARRIVE^27^	2018	Multination (7countries)	White (97.8) Other (2.2)	6,270/6,276	63.9/63.9	70.5/70.4	5.0 (median)	100 mg/d	HTN (64.8), hypercholesterolemia (58.2)
ASPREE^28^	2018	United States and Australia	White (91.3) Black (4.7) Hispanic (2.6) Other (1.4)	9,525/9,589	74.0 (median)	44.0/44.0	5.0	100 mg/d	HTN (74), DL (65), DM (11), CKD (26)
JPAD^29^	2008	Japan	Japanese	1,262/1,277	65.0/64.0	56.0/53.0	4.37	81 or 100 mg/d	HTN (58), DL (53), DM (100)
JPPP^30^	2014	Japan	Japanese	7,220/7,244	70.6/70.5	42.3/42.4	5.01/5.02	100 mg/d	HTN (84.9), DL (71.9), DM (33.9)

AAA = Aspirin for Asymptomatic Atherosclerosis; ABI = ankle-brachial index; ARRIVE = Aspirin to Reduce Risk of Initial Vascular Events; ASCEND = A Study of Cardiovascular Events in Diabetes; ASPREE = Aspirin in Reducing Events in the Elderly; CKD = chronic kidney disease; DL = dyslipidemia; DM = diabetes mellitus; HOT = Hypertension Optimal Treatment; HTN = hypertension; JPAD = Japanese Primary Prevention of Atherosclerosis with Aspirin for Diabetes; JPPP = Japanese Primary Prevention Project; POPADAD = Prevention of Progression of Arterial Disease and Diabetes; PPP = Primary Prevention Project; WHS = Women’s Health Study.

a The proportions of taking the following medications: diuretics, beta-blocker, nitrate, calcium-channel blocker, angiotensin-converting enzyme inhibitor or angiotensin II antagonist.

### Quality assessment and risk of bias

We assessed the quality and bias of the selected articles using the Cochrane Collaboration’s tool for assessing the risk of bias in randomized trials. The results of the risk of bias in the 11 eligible studies (Supplemental Table 2) were such that 3 studies^22^^,^^29^^,^^30^ had a high risk of blinding, and the HOT (Hypertension Optimal Treatment) study^20^ did not clearly describe the outcome data. Furthermore, because the control group in the JPPP (Japanese Primary Prevention Project) study^30^ was not treated with any placebo or medication, blinding bias was unavoidable. The remaining 2 studies^22^^,^^29^ were included in the final analysis because they did not have a significant impact on the results after being removed through sensitivity analysis. The level of evidence for each outcome ranged from very low (MI event) to low (CV death and ICH). The details of GRADE and a summary of the outcomes from the enrolled RCTs are shown in Supplemental Table 3.

The *P* value of Egger’s test to assess publication bias was higher than 0.05 for all main outcomes. Therefore, we conclude that there was no publication bias (Supplemental Table 4). The filled RRs calculated by the trim-and-fill method were almost similar to the pooled RRs, which could also be supported by the absence of publication bias (Supplemental Table 4). Funnel plots with the trim-and-fill method are presented in Supplemental Figures 1 to 9.

### Effect of aspirin for CV outcome and major bleeding

**Figure 2:**
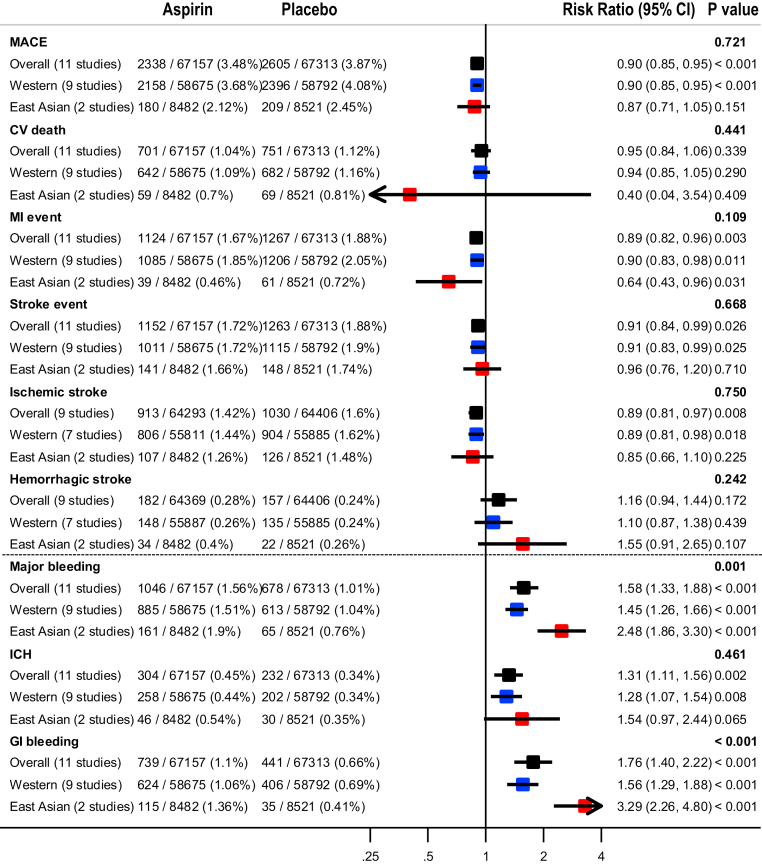
Outcomes Between Aspirin and Placebo Across Westerners and East Asians The black, blue, and red squares indicate the cardiovascular (CV) outcome for overall, Western, and East Asian populations, respectively. The bold *P* values indicated the difference of CV outcomes between the Western and East Asian populations. A major adverse cardiovascular event (MACE) indicated CV death, myocardial infarction (MI), and stroke. The 2 studies (PPP [Primary Prevention Project] and POPADAD [Prevention of Progression of Arterial Disease and Diabetes]) did not suggest detailed information regarding ischemic or hemorrhagic stroke. GI = gastrointestinal; ICH = intracranial hemorrhage.

**Figure 3:**
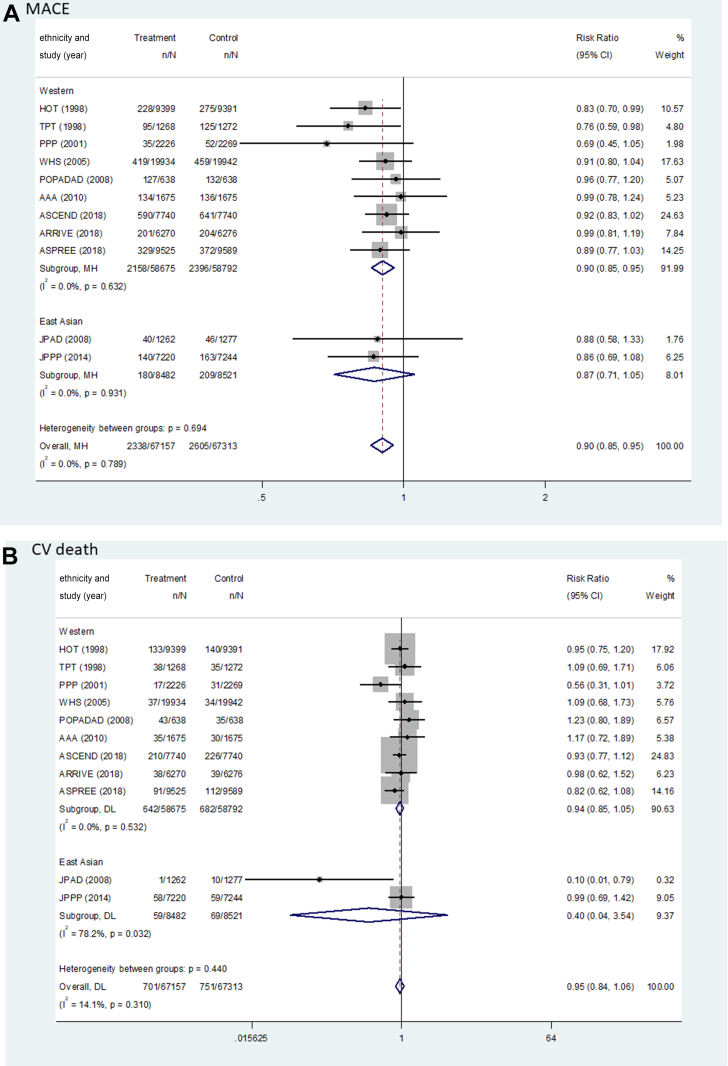

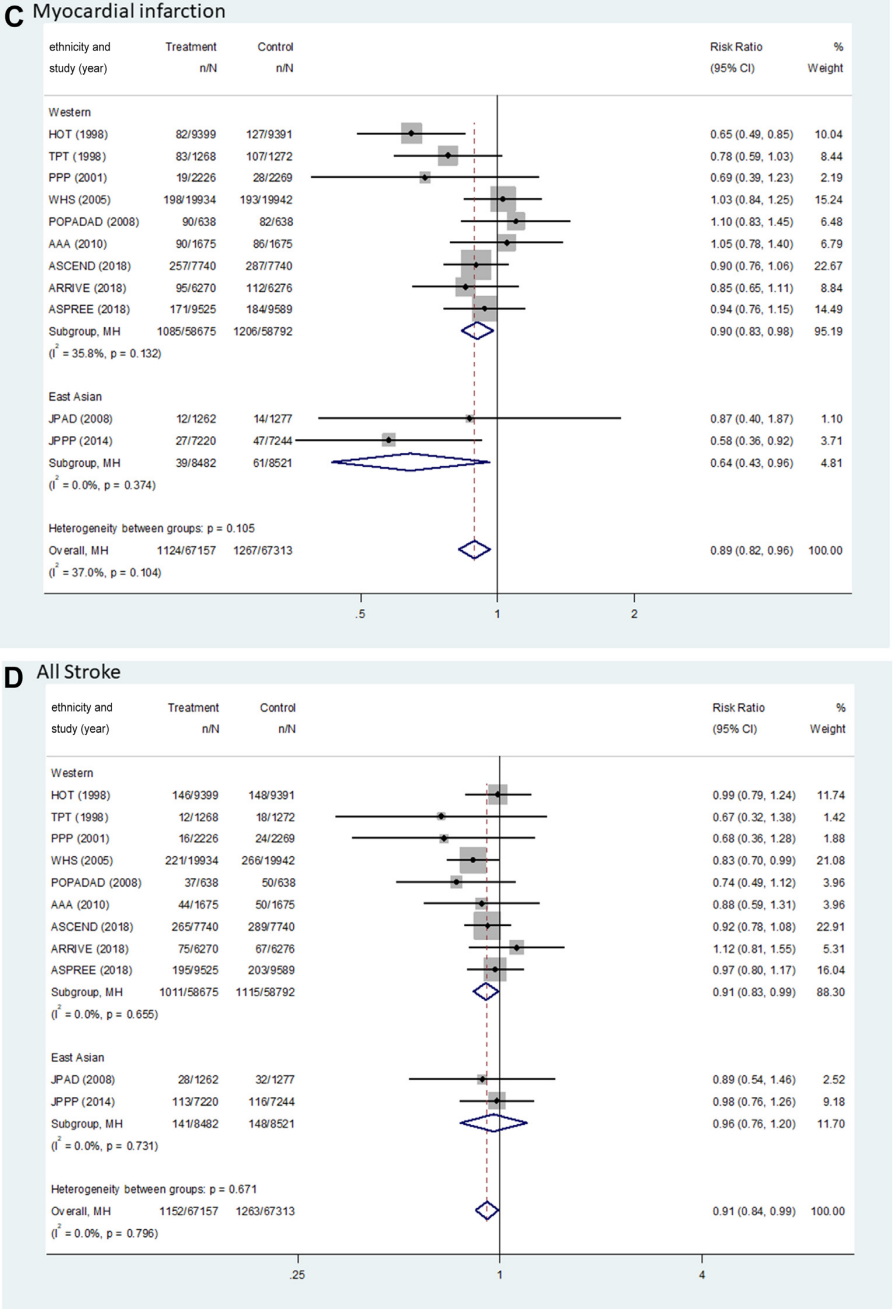

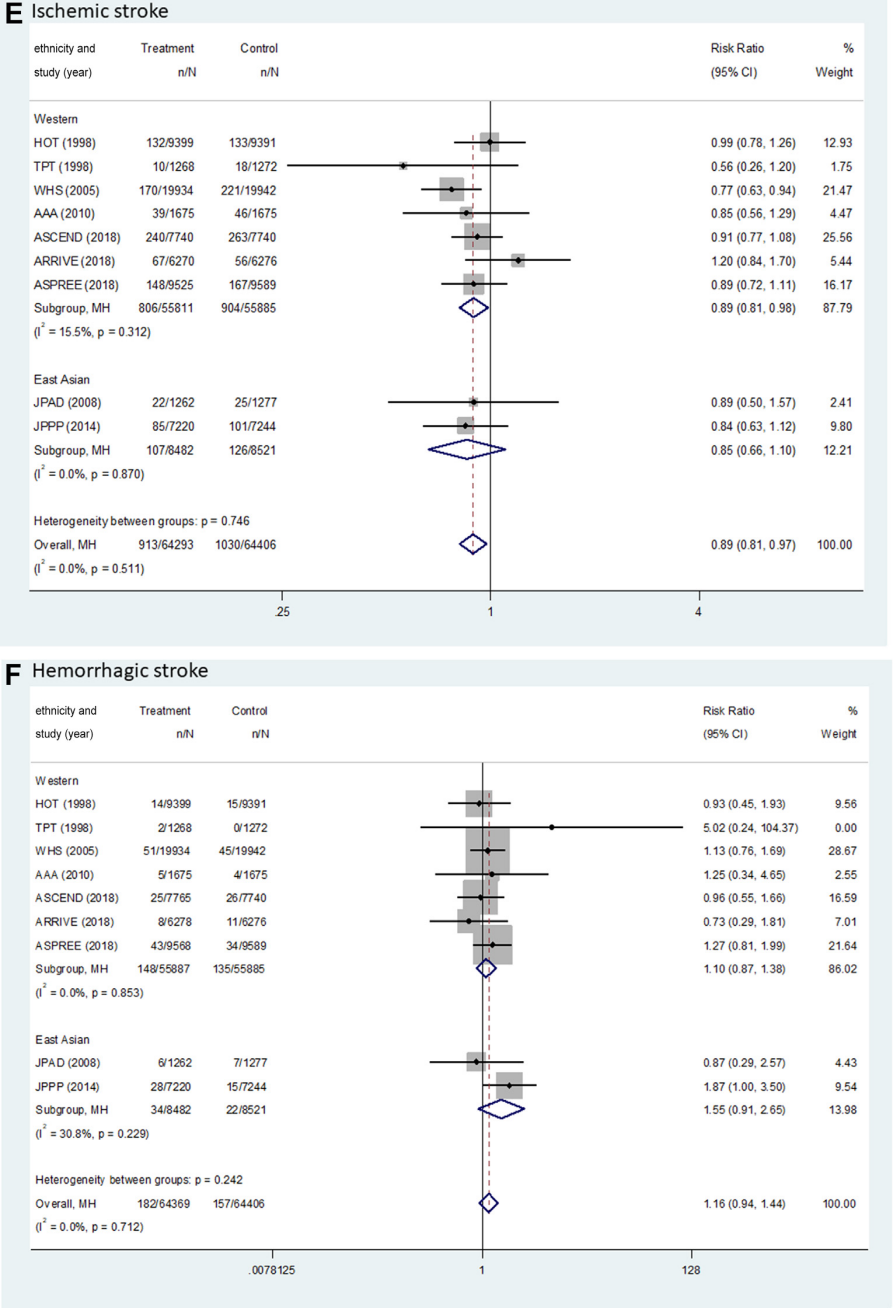
Forest Plots Regarding Efficacy of Aspirin Between Westerners and East Asians MACE indicated CV death, MI, and stroke. AAA = Aspirin for Asymptomatic Atherosclerosis; ARRIVE = Aspirin to Reduce Risk of Initial Vascular Events; ASCEND = A Study of Cardiovascular Events in Diabetes; ASPREE = Aspirin in Reducing Events in the Elderly; HOT = Hypertension Optimal Treatment; JPAD = Japanese Primary Prevention of Atherosclerosis with Aspirin for Diabetes; JPPP = Japanese Primary Prevention Project; WHS = Women’s Health Study; other abbreviations as in Figure 2.

**Figure 4:**
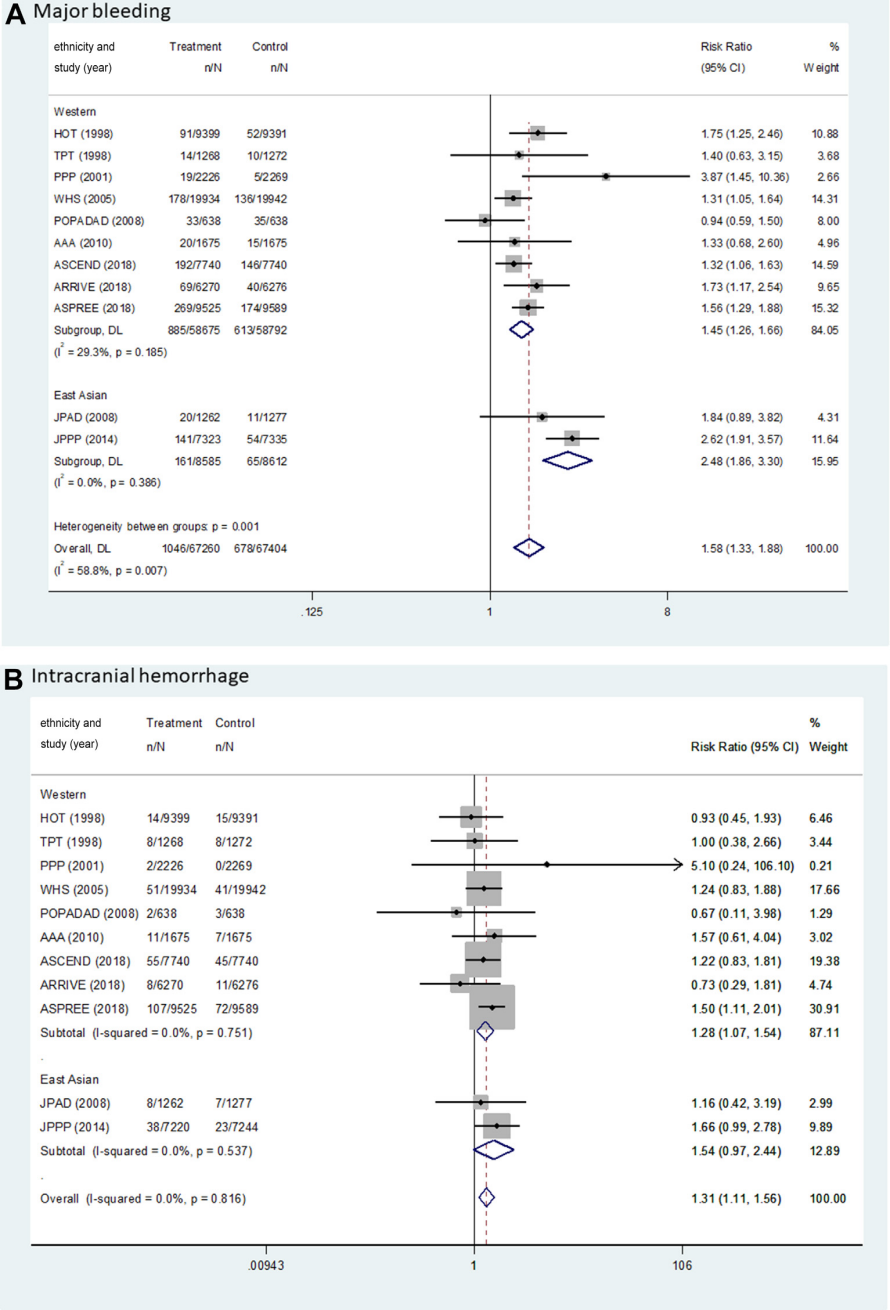

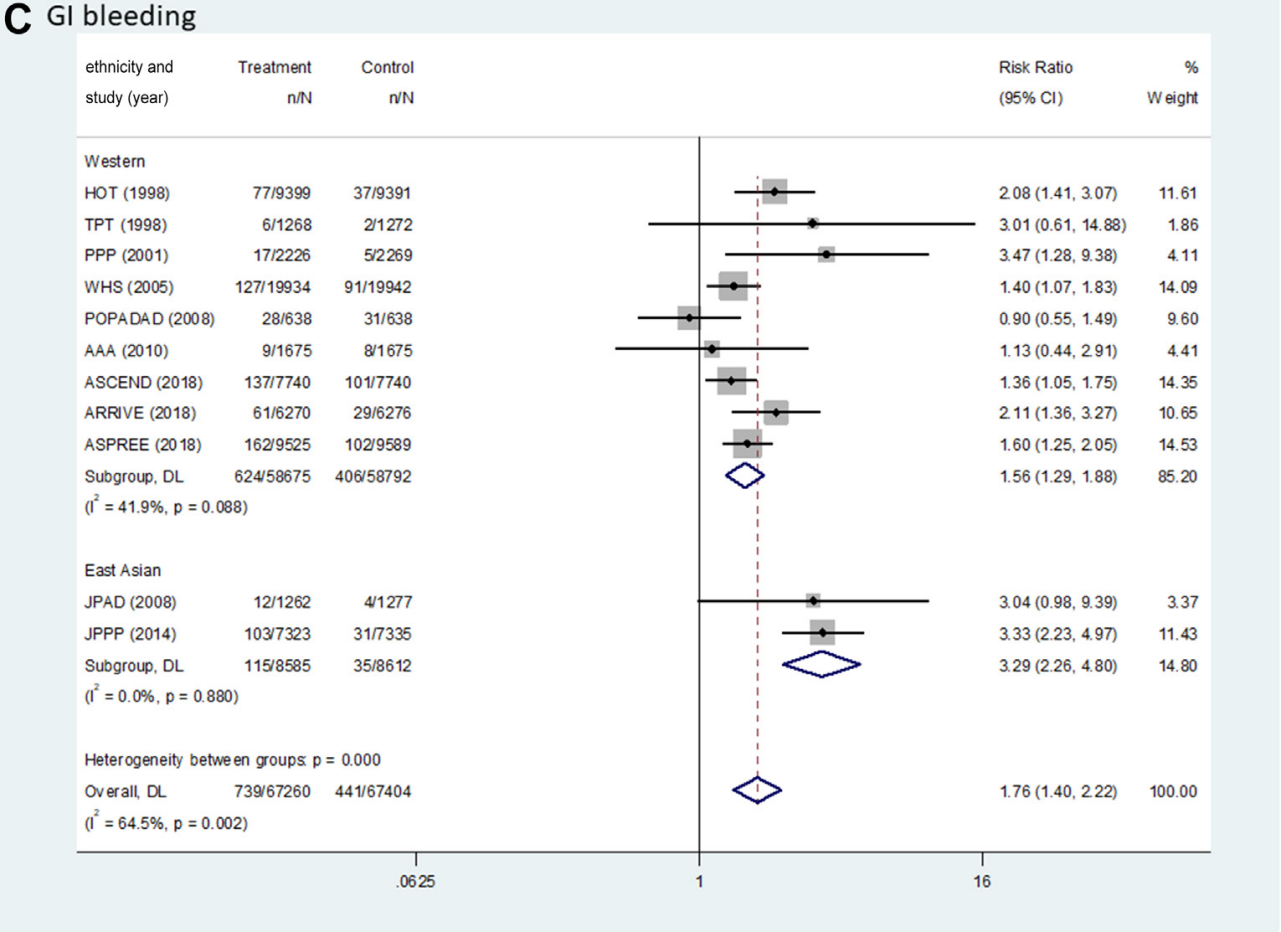
Forest Plots Regarding Safety of Aspirin Between Westerners and East Asians Major bleeding indicated ICH and GI bleeding. Abbreviations as in Figures 2 and 3.

Figure 2 and Table 2 show the pooled results (RR, RD, and number needed to treat) of the primary outcomes. The beneficial effect of aspirin was shown in terms of MACEs (RR: 0.90; 95% CI: 0.85-0.95; *P <* 0.001; *I*^2^ = 0.0%; *P* for heterogeneity = 0.789), which was related to a reduction in MI (RR: 0.89; 95% CI: 0.82-0.96; *P =* 0.003, *I*^2^ = 37.0%; *P* for heterogeneity = 0.104) and ischemic stroke (RR: 0.89; 95% CI: 0.81-0.97; *P =* 0.008; *I*^2^ = 0.0%; *P* for heterogeneity = 0.511) (Figure 3). There was no significant difference in the rate of CV death between the groups (RR: 0.95; 95% CI: 0.84-1.06; *P =* 0.339; *I*^2^ = 14.1%; *P* for heterogeneity = 0.310). The harmful outcome of aspirin was significantly higher for major bleeding (RR: 1.58; 95% CI: 1.33-1.88; *P <* 0.001; *I*^2^ = 58.8%; *P* for heterogeneity = 0.007), which was associated with an increased rate of ICH (RR: 1.31; 95% CI: 1.11-1.56; *P =* 0.002; *I*^2^ = 0.0%; *P* for heterogeneity = 0.816) and major GI bleeding (RR: 1.76; 95% CI: 1.40-2.22; *P <* 0.001; *I*^2^ = 64.5%; *P* for heterogeneity = 0.002) (Figure 4).

**Table 2:** Pooled Risk Difference and Number Needed to Treat of Benefit or Harm for Cardiovascular Outcomes

	Western Population		East Asian Population	
	Pooled Risk Difference per 1,000 Persons (95% CI)	NNTB or NNTH	Pooled Risk Difference per 1,000 Persons (95% CI)	NNTB or NNTH
MACE	−3.97 (−6.18 to −1.77)	251.6	−3.31 (−7.80 to 1.19)	302.5
CVD death	−0.66 (−1.87 to 0.55)	1,518.4	−1.87 (−3.43 to −0.31)	534.9
MI event	−2.02 (−3.60 to −0.44)	494.7	−2.56 (−4.86 to −0.26)	390.5
Stroke event	−1.73 (−3.26 to −0.21)	576.5	−0.75 (−4.63 to 3.14)	1,341.5
Ischemic stroke	−1.73 (−3.17 to −0.29)	576.5	−2.17 (−5.67 to 1.32)	460.4
Hemorrhagic stroke	0.24 (−0.35 to 0.83)	4,234.9	1.43 (−0.30 to 3.15)	701.0
Major bleeding	4.69 (3.37-5.94)	213.2	11.35 (7.91-14.79)	88.1
ICH	0.96 (0.21-1.65)	1,040.3	1.90 (−0.10 to 3.91)	525.6
GI bleeding	3.73 (2.66-4.80)	268.2	9.45 (6.64-12.26)	105.8

CVD = cardiovascular disease; GI = gastrointestinal; ICH = intracranial hemorrhage; MACE = major adverse cardiovascular event(s); MI = myocardial infarction; NNTB = number needed to treat to benefit; NNTH = number needed to treat to harm.

### Effect of aspirin between East Asian vs Western population

For MACEs, there was no heterogeneity between the East Asian (RR: 0.87; 95% CI: 0.71-1.05; *P =* 0.151; pooled RD: −3.31; NNTB: 302.5) and Western populations (RR: 0.90; 95% CI: 0.85-0.95; *P <* 0.001; pooled RD: −3.97; NNTB: 251.6) (*P*_interaction_ = 0.721) (Figure 2, Table 2). In East Asian individuals, the aspirin group showed a lower rate of only MI events compared with the control group (RR: 0.64; 95% CI: 0.43-0.96; *P =* 0.031; pooled RD: −2.56; NNTB: 390.5). Among Western population, low-dose aspirin compared with control was associated with decreased risks of MACEs, MI (RR: 0.90; 95% CI: 0.83-0.98; *P =* 0.011; pooled RD: −2.02; NNTB: 494.7), and ischemic stroke (RR: 0.89; 95% CI: 0.81-0.98; *P =* 0.018; pooled RD: −1.73; NNTB: 576.5), respectively.

For major bleeding, potential heterogeneity was noted between East Asian (RR: 2.48; 95% CI: 1.86-3.30; *P <* 0.001; pooled RD: 11.35; NNTH: 88.1) and Western populations (RR: 1.45; 95% CI: 1.26-1.66; *P <* 0.001; pooled RD: 4.69; NNTH: 213.2) (*P*_interaction_ = 0.001) (Figure 2, Table 2). Low-dose aspirin, compared with control, was associated with a significant increased risk of major GI bleeding in trials enrolling an East Asian population (RR: 3.29; 95% CI: 2.26-4.80; *P <* 0.001; pooled RD: 9.45; NNTH: 105.8) compared to trials with predominantly non–East Asian populations (RR: 1.56; 95% CI: 1.29-1.88; *P <* 0.001; pooled RD: 3.73; NNTH: 268.2) (*P*_interaction_ < 0.001).

**Figure 5:**
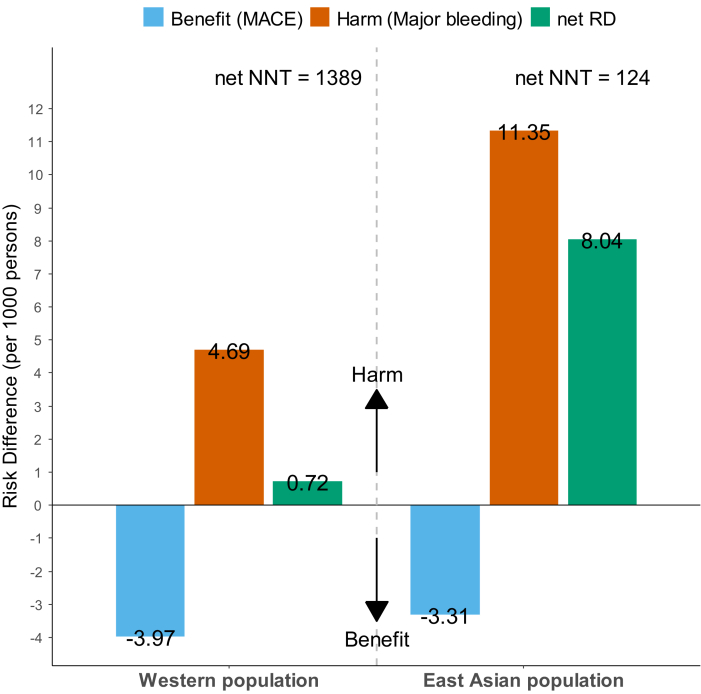
The Net RD and Net NNT The blue bar and negative value are the benefits of major adverse cardiovascular events (MACEs), which were 3.97 or 3.31 persons per 1,000 persons less occurred in the aspirin arm compared with the control arm. The orange bar and positive value are the harms of major bleeding, which were 4.66 or 11.35 persons per 1,000 persons more occurred in the aspirin arm. The green bar and value were net risk differences (RDs), which were calculated by benefit + harm values. The net number needed to treat (NNT) calculated by: 1 / (net RD).

The net RDs (RD of MACEs plus RD of major bleeding) between both groups were 0.72 and 8.04 per 1,000 persons in Western and East Asian individuals, respectively (Figure 5). The net number needed to treat (NNTB of MACEs plus NNTH of major bleeding) was 1,389 in the Western population and 124 in the Asian population, indicating that there will be 1 additional harmful effect more than a beneficial effect for every 1,389 or 124 subjects treated, respectively.

### Sensitivity analysis

The leave-one-out method in sensitivity analyses for MACEs, CV death, hemorrhagic stroke, GI bleeding, and major bleeding showed similar pooled RRs. However, when some studies^23^^,^^28^ were excluded, the ischemic stroke and ICH events were not statistically significant. Furthermore, the effect size of the pooled RRs was not large compared with the results of all the included studies (Supplemental Tables 5 and 6).

## Discussion

To the best of our knowledge, none of the meta-analysis studies of RCTs regarding the primary preventive effect of aspirin across the ethnicity was performed until now (Central Illustration). The present analysis including individuals without symptomatic CVD demonstrated that: 1) the use of low-dose aspirin showed a similar benefit in reducing CV events among both populations; 2) aspirin treatment significantly increased the risk of major bleeding, particularly driven by major GI bleeding, in East Asian individuals compared to Westerners; and 3) the primary preventive effect of low-dose aspirin in all populations had more harm than benefit, which was pronounced in the East Asian population.

**Central Illustration:**
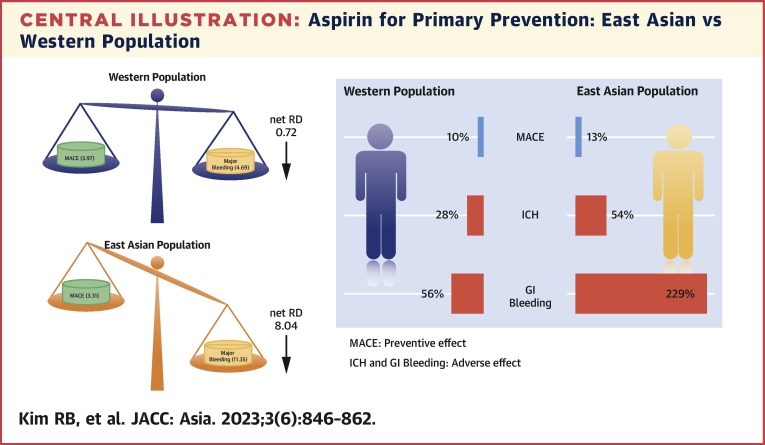
Aspirin for Primary Prevention: East Asian vs Western Population We searched randomized clinical trials (RCTs) for intervention with low-dose aspirin (≤100 mg once daily) in participants without symptomatic cardiovascular disease until December 31, 2021. Two RCTs included 17,003 East Asians, and 9 RCTs had 117,467 Western participants. Aspirin treatment showed a similar effect in reducing the rate of a major adverse cardiovascular event (MACE) (relative risk [RR] of East Asians: 0.87; 95% CI: 0.71-1.05; RR of Westerners: 0.90; 95% CI: 0.85-0.95) (*P*_interaction_ = 0.721). In contrast, the risk of major bleeding during aspirin vs control was greater in the East Asian population (RR: 2.48; 95% CI: 1.86-3.30) compared with the Western population (RR: 1.45; 95% CI: 1.26-1.66) (*P*_interaction_ = 0.001), which was driven by more frequent gastrointestinal (GI) bleeding (RR of East Asians: 3.29; 95% CI: 2.26-4.80 vs RR of Westerners: 1.56; 95% CI: 1.29-1.88) (*P*_interaction_ < 0.001). The net relative differences (RDs) (RD of MACE plus RD of major bleeding) were 8.04 and 0.69 per 1,000 persons in East Asian and Western participants, indicating 124 and 1,449 of the net number needed to harm, respectively. Therefore, low-dose aspirin for primary prevention in East Asians must be cautiously prescribed because of an increased risk of major bleeding relative to Western counterparts. ICH = intracranial hemorrhage.

Since 2006, there have been many meta-analyses of RCT studies looking at primary preventive effects of aspirin on populations without previous CV events.^2^, ^3^, ^4^^,^^31^^,^^32^ Most of the RCTs included in the present study were similar to those included in previous meta-analyses; however, most previous meta-analyses included the BDT (British Doctors Trial)^33^ and PHS (Physicians’ Health Study).^34^ We did not include these studies because the dose of aspirin (500 mg/d and 325 mg per alternate day) was different from our criteria of low-dose aspirin (≤100 mg once daily).

The results from most previous meta-analyses are similar to the findings of our study; the risk of MACEs, MI, and ischemic stroke were reduced. However, the absolute reduction in risk was minimal. Furthermore, the harmful effects of bleeding were greater than the beneficial effects. In the 2009 ATT (Antithrombotic Trialists’) collaboration,^2^ the absolute rate difference in the reduction of serious vascular events was 0.06%, whereas that of major GI and extracranial bleeding events was 0.3% higher in the aspirin vs the control group. In the 2019 meta-analysis from Zheng and Roddick,^31^ the absolute difference of aspirin treatment in major CV outcomes was 4.3 per 10,000 participant-years, whereas its difference in major bleeding was 6.7 per 10,000 participant-years. Therefore, it can be said that the beneficial effects of aspirin for the primary prevention of CV events is unclear.

Epidemiologic studies have demonstrated marked variations in the prevalence and natural history, demographics, CV risk factors, and CVD outcomes across the ethnicities.^35^ The precision medicine for CVD prophylaxis must consider individual gene variability, pharmacokinetics, pharmacodynamics, demographic and environmental data, and CV risk profiles to maximize efficacy while minimizing adverse events.^14^ Accordingly, ethnicity remains associated with differing rates of CVD. The East Asian population has shown a lower incidence of CAD and a decreased risk of post-PCI atherothrombotic complications compared with the Caucasian population.^13^^,^^36^ Although the underlying mechanisms of this observation should be multifactorial and complex, the low thrombogenic profile (eg, low levels of inflammation and coagulation activity and low incidence of obesity) shown in East Asian individuals may be a crucial factor to account for their unique property.

Oral antithrombotic therapy is a key element of CV pharmacotherapy for primary and secondary prevention of CVD. The results of previous studies could not confirm the difference between the beneficial and harmful effects of antithrombotic therapy according to ethnicity. East Asian patients had a significantly higher risk of hemorrhagic events when given dual antiplatelet therapy for secondary prevention after coronary intervention in patients with significant CAD.^37^ In patients with nonvalvular atrial fibrillation, the ICH risk in East Asian patients still appeared to be relatively higher during anticoagulant treatment compared with non-Asian patients.^38^ The present analysis demonstrates that the benefit of low-dose aspirin for primary prevention in East Asian individuals may not be expected because of the limited benefit on ischemic events and increased harm on major bleeding.

There are many reasons for the difference in the effect of aspirin between the ethnicities. However, there are several suggested mechanisms why East Asians have a higher bleeding tendency during antithrombotic treatment than Western counterparts. First, the epidemiologic or demographic disparity between the ethnicities may explain partly a greater bleeding tendency in East Asians relative to their Western counterparts. It is well-known that *Helicobacter pylori* infection has a higher incidence in East Asians than in Western populations (50%-70% in East Asians vs 30%-50% in Caucasians).^39^^,^^40^ This infection can cause gastric erosion and peptic ulcer related with GI bleeding, and more virulent strains of *Helicobacter pylori* in East Asian populations could further increase the risk of GI bleeding in patients taking aspirin.^40^ Genetic polymorphisms affect the efficacy of treatment using proton pump inhibitors (PPIs), which can influence local rates of *H. pylori* resistance. In addition, intracranial atherosclerosis (30%-50% in East Asians vs 15%-30% in Caucasians) and poststroke hemorrhagic transformation are more prevalent among East Asians compared with Caucasians.^13^ Therefore, East Asians have a higher prevalence of ICH (and lacunar strokes) compared with ischemic stroke (30% and 70%, respectively) in comparison with Whites (15% and 85%, respectively).^14^ Second, the East Asian population has a unique responsiveness to antithrombotic agents because of pharmacogenetics and low body weight.^13^^,^^36^ Most antithrombotic agents show enhanced pharmacokinetic and pharmacodynamic profiles in East Asian vs Caucasian subjects, except for clopidogrel and edoxaban.^13^ For aspirin, there are no confirmative experimental data to show the interethnic difference in laboratory responsiveness to aspirin. In addition, demographic differences, such as diet and body weight, between the ethnicities could affect the difference in the maintenance time in the therapeutic range for antithrombotic agents. For example, the time taken to reach the therapeutic range of aspirin was lower in East Asians (36.0%) than in North Americans (50.9%) and Western Europeans (62.4%).^13^^,^^41^ An aspirin dose of 75 mg daily reduced the risk of GI bleeding by about 30% compared with 150 mg daily.^42^

The occurrence of peptic ulcer disease is attributed to many etiologies, such as *H. pylori* infection, drug use (eg, nonsteroidal anti-inflammatory drugs and aspirin), alcohol, smoking, stress, lifestyle habits, and genetic characteristics.^39^^,^^40^ Compared with the Caucasian population, the East Asian population shows an increased frequency of upper GI injuries related with a higher incidence of *H. pylori* infection and drug-related complications.^13^ Therefore, identifying high-risk populations (eg, old age and comorbidities) and applying the gastroprotective strategy can be more important for the East Asian population during aspirin treatment. Compared with other acid suppressant drugs, PPIs have shown the most effective gastroprotective potential during nonsteroidal anti-inflammatory drug or antithrombotic treatment. Because East Asian individuals have shown high-risk bleeding on aspirin treatment, its use for primary prevention must be cautious, and a default use with PPI should be considered in high-risk populations.^13^ However, there are remaining issues regarding durations and doses of PPIs.

In the recent major Western guidelines, aspirin is no longer recommended for primary prophylactic use. In the 2016 European guidelines on CVD prevention in clinical practice,^43^ primary prevention with antiplatelet therapy such as aspirin was not recommended in individuals without CVD because of the increased risk of major bleeding. The 2019 American College of Cardiology/American Heart Association Guidelines on Primary Prevention of Cardiovascular Disease^44^ reported that aspirin should be used infrequently in routine primary prevention because of a lack of net benefit. In addition, the recommendation statement of aspirin use to prevent CVD from the 2022 USPSTF^45^ recommended against initiating low-dose aspirin treatment for the primary prevention of CVD in adults aged 60 years or older. Given the findings in the current meta-analysis, the usage of aspirin for primary prevention among East Asians should be recommended against in clinical practice.

### Study limitations

First, the number of studies is not well balanced between East Asians and Westerners. Because only 2 East Asian RCTs including only a Japanese population were used for analysis, the results may be underpowered to observe the difference of CV outcome because of a suspected lower event rate. Second, this meta-analysis did not suggest the results according to age group and CVD risk because of the inability to specify these variables. According to the current guidelines,^45^ aspirin can be carefully taken at a certain age with high-risk CVD (eg, 40-59 years with a 10-year CVD risk of 10% or more). However, these problems may be insignificant because the age of the participants included in the analysis was mostly around their 50s or 60s. Third, the HOT study^20^ was conducted in 26 countries with some of the participants being Asians. Furthermore, some of the other studies^23^^,^^26^ also included Asians. However, this fact may not be a big issue because the proportion of Asian participants among these studies was just 1.1% to 1.4%. Additionally, there would be considerable differences in the frequency of statin and gastric antacid coprescriptions because the included RCTs were conducted over a long period between the 1990s and 2010s. These treatments can influence the efficacy and safety of aspirin, but most of the included studies did not report this information. Finally, the bleeding outcomes can occur by various causes such as trauma, hypertension, other drugs, and so on. However, this systematic review could not indicate the detailed information. Therefore, the bleeding events related with aspirin may be overestimated. The results of this study should be cautiously interpreted, taking in this lack of information.

## Conclusions

The benefit of low-dose aspirin for the primary prevention of CV events was limited in the East Asian population. During aspirin treatment, East Asians showed a similar benefit in reducing ischemic CV events and increased harm in relation to major bleeding, particularly increasing the risk of GI bleeding. In this population, the harm may be much greater than the benefit because of their greater bleeding tendency than the Western population. Therefore, it is difficult to recommend taking aspirin for the primary prevention of CV events in healthy East Asian adults or individuals without symptomatic CVD.

Perspectives **COMPETENCY IN PATIENT CARE:** East Asian individuals have shown different risk profiles for both thrombophilia and bleeding than their Western counterparts. Among PCI-treated patients on dual antiplatelet therapy with a potent P2Y_12_ inhibitor and aspirin, East Asian patients have shown an increased risk of serious bleeding compared with Western subjects. Is there a difference between the East Asian and Western populations in the primary preventive effect of low-dose aspirin (≤100 mg once daily)?

**TRANSLATIONAL OUTLOOK:** In this systematic meta-analysis including 11 randomized clinical trials, low-dose aspirin treatment showed a similar effect in reducing the risk of ischemic events. However, the increased rate of major bleeding with low-dose aspirin was significantly greater in the East Asian population compared with the Western population, driven by a trend toward more frequent GI bleeding. Prescribing aspirin for primary prevention appeared more harmful than beneficial in East Asian individuals.

## Funding Support and Author Disclosures

This study was supported by the Science Research Program through the CAU Thrombosis and Biomarker Center funded by the Chung-Ang University Gwangmyeong Hospital. The content is solely the responsibility of the authors and does not necessarily represent the official views of any funding agencies. Dr Gurbel has received grants and personal fees from Bayer HealthCare, Otitopic, Amgen, Janssen, and US World-Meds; has received grants from Instrumentation Laboratory, Hikari Dx, Haemonetics, Medicure, and Idorsia Pharmaceuticals; has received personal fees from UpToDate; and has patents “Detection of Restenosis Risk in Patients Issued” and “Assessment of Cardiac Health and Thrombotic Risk in a Patient.” Dr Jeong has received honoraria for lectures from AstraZeneca, Daiichi Sankyo, Sanofi, Han-mi Pharmaceuticals, and Yuhan Pharmaceuticals; and has received research grants or support from Yuhan Pharmaceuticals and U and I Corporation. All other authors have reported that they have no relationships relevant to the contents of this paper to disclose.
