# Supplementary material for: Low-Dose Aspirin for Primary Prevention of Cardiovascular Events Comparing East Asians With Westerners: A Meta-Analysis
Source: JACC Asia. 2023 Sep 12;3(6):846–62. doi: 10.1016/j.jacasi.2023.07.008 (PMC10751647; doi:10.1016/j.jacasi.2023.07.008)
Supplement: Supplemental Tables 1-6 and Supplemental Figures 1-9 [file mmc1.docx]

**Supplemental Table 1. The Search Terms at Each Electronic Databases**

1. **PubMed search term**

#1 Prevent*

#2 Prophylaxis

#3 (#1 OR #2)

#4 Aspirin* [TIAB]

#5 Acetylsalicylic acid [TIAB]

#6 (#4 OR #5)

#7 Placebo*

#8 Control group*

#9 (#7 OR #8)

#10 Cardiovascular diseases [MeSH]

#11 Cerebrovascular disorders [MeSH]

#12 Hemorrhage [MeSH]

#13 (#10 OR #11 OR #12)

#14 Clinical [TIAB] AND trial [TIAB]

#15 Clinical trials as topic [MeSH]

#16 Clinical trial [PT]

#17 Random* [TIAB]

#18 (#14 OR #15 OR #16 OR #17)

#19 (#3 AND #6 AND #9 AND #13 AND #18)

**Number of articles: 1,450** (to 2021/12/31, applied filter: Clinical trial, RCT, Human)

1. **EMBASE search term**

('prevention and control'/exp OR 'prophylaxis'/exp) AND ('acetylsalicylic acid'/exp OR 'acetylsalicylic acid' OR aspirin:ti,ab) AND ('placebo'/exp OR 'control'/exp OR 'control group') AND ('cardiovascular disease'/exp OR 'cerebrovascular disease'/exp OR 'bleeding'/exp) AND ('clinical trial (topic)'/exp OR 'randomized controlled trial'/exp OR 'randomization'/exp)

**Number of articles: 1,403** (human, to 2021year)

1. **Cochrane Library search term**

#1 prevention

#2 prophylaxis

#3 (#1 OR #2)

#4 (aspirin): ti,ab

#5 (acetylsalicylic acid): ti,ab

#6 (#4 OR #5)

#7 placebo

#8 control group

#9 (#7 OR #8)

#10 MeSH: [cardiovascular diseases] explode

#11 MeSH: [cerebrovascular disorders] explode

#12 MeSH: [Hemorrhage] explode

#13 (#10 OR #11 OR #12)

#14 clinical trial

#15 (random*): ti,ab

#16 (clinical trial*): ti,ab

#17 (#14 OR #15 OR #16)

#18 (#3 AND #6 AND #9 AND #13 AND #16)

**Number of articles: 1,324** (to 2021/Dec/31)

**Supplemental Table 2.** **Quality Assessment of Selected Studies by Cochran’s Risk of Bias Tool**

| **Study** | **Year** | **Random sequence generation** | **Allocation concealment** | **Blinding of participants and personnel** | **Blinding of outcome assessment** | **Incomplete outcome data** | **Selective reporting** | **Other bias** |
| --- | --- | --- | --- | --- | --- | --- | --- | --- |
| HOT^20^ | 1998 | Low | Low | Low | Low | **Unclear** | Low | Low |
| TPT^21^ | 1998 | Low | Low | Low | Low | Low | Low | Low |
| PPP^22^ | 2001 | Low | Low | **High** | Low | Low | Low | Low |
| WHS^23^ | 2005 | Low | Low | Low | Low | Low | Low | Low |
| POPADAD^24^ | 2008 | Low | Low | Low | Low | Low | Low | Low |
| AAA^25^ | 2010 | Low | Low | Low | Low | Low | Low | Low |
| ASCEND^26^ | 2018 | Low | Low | Low | Unclear | Low | Low | Low |
| ARRIVE^27^ | 2018 | Low | Low | Low | Low | Low | Low | Low |
| ASPREE^28^ | 2018 | Low | Low | Low | Low | Low | Low | Low |
| JPAD^29^ | 2008 | Low | Low | **High** | Low | Low | Low | Low |
| JPPP^30^ | 2014 | Low | Low | **High** | Low | Low | Low | Low |

**Abbreviations:** AAA, the Aspirin for Asymptomatic Atherosclerosis trial; ARRIVE, the Aspirin to Reduce Risk of Initial Vascular Events study; ASCEND, A Study of Cardiovascular Events in Diabetes; ASPREE, the Aspirin in Reducing Events in the Elderly trial; HOT, the Hypertension Optimal Treatment Study; JPAD, the Japanese Primary Prevention of Atherosclerosis with Aspirin for Diabetes Trial; JPPP, the Japanese Primary Prevention Project; POPADAD, the Prevention of Progression of Arterial Disease and Diabetes trial; PPP, the Primary Prevention Project; WHS, the Women’s Health Study.

**Supplemental Table 3.** **GRADE and Summary of Outcomes for Low-dose Aspirin from the All Studies**

| **Outcomes** | **Anticipated absolute effects**^a^  **(95% CI)** | | **Relative effect,**  **RR (95% CI)** | **No of participants (studies)** | **Certainty of the evidence (GRADE)**^b^ |
| --- | --- | --- | --- | --- | --- |
|  | **Risk with Placebo** | **Risk with Aspirin** |  |  |  |
| **MACE** | **39 per 1,000** | **35 per 1,000**  **(33 to 37)** | **0.90**  **(0.85 to 0.94)** | **134,470**  **(11 RCTs)** | **⨁⨁⨁◯**  **Moderate** |
| CV death | 11 per 1,000 | 10 per 1,000  (9 to 12) | 0.95  (0.84 to 1.06) | 134,470  (11 RCTs) | ⨁⨁◯◯  Low |
| MI event | 19 per 1,000 | 17 per 1,000  (15 to 18) | 0.89  (0.82 to 0.96) | 134,470  (11 RCTs) | ⨁◯◯◯  Very low |
| Stroke events | 19 per 1,000 | 17 per 1,000  (16 to 19) | 0.91  (0.84 to 0.99) | 134,470  (11 RCTs) | ⨁⨁⨁◯  Moderate |
| Ischemic stroke^c^ | 16 per 1,000 | 14 per 1,000  (13 to 16) | 0.89  (0.81 to 0.97) | 128,699  (9 RCTs) | ⨁⨁⨁◯  Moderate |
| Hemorrhagic stroke^c^ | 2 per 1,000 | 3 per 1,000  (2 to 4) | 1.16  (0.94 to 1.44) | 128,775  (9 RCTs) | ⨁⨁⨁◯  Moderate |
| **Major bleeding** | **10 per 1,000** | **16 per 1,000**  **(13 to 19)** | **1.58**  **(1.33 to 1.88)** | **134,664**  **(11 RCTs)** | **⨁⨁⨁◯**  **Moderate** |
| ICH | 3 per 1,000 | 5 per 1,000  (4 to 6) | 1.31  (1.11 to 1.56) | 134,664  (11 RCTs) | ⨁⨁◯◯  Low |
| GI bleeding | 7 per 1,000 | 12 per 1,000  (9 to 14) | 1.76  (1.40 to 2.22) | 134,664  (11 RCTs) | ⨁⨁⨁◯  Moderate |
| **Abbreviations:** CV, cardiovascular disease; MACE, major adverse cardiovascular events; MI, myocardial infarction; ICH, intracranial hemorrhage; GI, gastrointestinal; GRADE, Grading of Recommendations, Assessment, Development and Evaluation; CI, confidence interval; HR, odds ratio.  ^a^The risk in the intervention group (and its 95% CI) is based on the assumed risk in the comparison group and the relative effect of the intervention (and its 95% CI). | | | | | |
| ^b^**GRADE Working Group grades of evidence**  **High certainty:** we are very confident that the true effect lies close to that of the estimate of the effect.  **Moderate certainty:** we are moderately confident in the effect estimate: the true effect is likely to be close to the estimate of the effect, but there is a possibility that it is substantially different.  **Low certainty:** our confidence in the effect estimate is limited: the true effect may be substantially different from the estimate of the effect.  **Very low certainty:** we have very little confidence in the effect estimate: the true effect is likely to be substantially different from the estimate of effect.  ^c^The PPP and POPADAD studies do not suggest the detailed information regarding ischemic or hemorrhagic stroke. | | | | | |

**Supplemental Table 4. Publication Bias Assessment by Trim and Filled Method and Egger's test**

| **Outcomes** | **Pooled RR (95% CI)** | **Trim and filled RR (95% CI)** | **p-value of Egger's test**^a^ |
| --- | --- | --- | --- |
| **MACE** | **0.90 (0.85, 0.95)** | **0.89 (0.84, 0.95)** | **0.261** |
| CV death | 0.95 (0.84, 1.06) | 0.93 (0.82, 1.04) | 0.313 |
| MI event | 0.89 (0.82, 0.96) | 0.89 (0.82, 0.96) | 0.254 |
| Stroke event | 0.91 (0.84, 0.99) | 0.91 (0.84, 0.99) | 0.307 |
| Ischemic stroke | 0.89 (0.81, 0.97) | 0.89 (0.81, 0.97) | 0.835 |
| Hemorrhagic stroke | 1.16 (0.94, 1.44) | 1.15 (0.93, 1.43) | 0.877 |
| **Major bleeding** | **1.58 (1.33, 1.88)** | **1.55 (1.29, 1.86)** | **0.494** |
| ICH | 1.31 (1.11, 1.56) | 1.29 (1.09, 1.54) | 0.619 |
| GI bleeding | 1.76 (1.40, 2.22) | 1.53 (1.18, 1.97) | 0.314 |
| **Abbreviations:** CV, cardiovascular disease; MACE, major adverse cardiovascular events; MI, myocardial infarction; ICH, intracranial hemorrhage; GI, gastrointestinal; GRADE, Grading of Recommendations, Assessment, Development and Evaluation; CI, confidence interval; HR, odds ratio.  ^a^If the p-value is greater than 0.05, it is judged that there is no publication bias. | | | |

**Supplemental Table 5 Results of leave-one-out method in sensitivity analysis for MACE, CV death, MI event, all Stroke event and Ischemic stroke outcomes**

|  | **MACE** | **CV death** | **MI event** | **Stroke event** | **Ischemic stroke** |
| --- | --- | --- | --- | --- | --- |
| **Study** | **RR [95% CI]** | **RR [95% CI]** | **RR [95% CI]** | **RR [95% CI]** | **RR [95% CI]** |
| Omitting HOT 1998 | 0.90 [0.85, 0.96] | 0.92 [0.82, 1.04] | 0.91 [0.84, 1.00] | 0.90 [0.83, 0.98] | 0.87 [0.79, 0.96] |
| Omitting TPT 1998 | 0.90 [0.85, 0.96] | 0.92 [0.82, 1.03] | 0.90 [0.82, 0.98] | 0.92 [0.84, 0.99] | 0.89 [0.81, 0.98] |
| Omitting PPP 2001 | 0.90 [0.85, 0.95] | 0.95 [0.85, 1.05] | 0.89 [0.82, 0.97] | 0.92 [0.85, 0.99] | 0.89 [0.81, 0.97] |
| Omitting WHS 2005 | 0.89 [0.84, 0.95] | 0.92 [0.82, 1.03] | 0.86 [0.79, 0.94] | 0.93 [0.85, 1.02] | 0.92 [0.83, 1.02] |
| Omitting POPADAD 2008 | 0.89 [0.84, 0.95] | 0.91 [0.82, 1.02] | 0.87 [0.80, 0.95] | 0.92 [0.85, 1.00] | 0.89 [0.81, 0.97] |
| Omitting AAA 2010 | 0.89 [0.84, 0.94] | 0.92 [0.82, 1.03] | 0.87 [0.80, 0.95] | 0.91 [0.84, 0.99] | 0.89 [0.81, 0.97] |
| Omitting ASCEND 2018 | 0.89 [0.83, 0.95] | 0.93 [0.82, 1.06] | 0.88 [0.81, 0.97] | 0.91 [0.83, 1.00] | 0.88 [0.79, 0.98] |
| Omitting ARRIVE 2018 | 0.89 [0.83, 0.94] | 0.93 [0.83, 1.03] | 0.89 [0.82, 0.97] | 0.90 [0.83, 0.98] | 0.87 [0.79, 0.95] |
| Omitting ASPREE 2018 | 0.90 [0.84, 0.95] | 0.95 [0.85, 1.07] | 0.88 [0.80, 0.96] | 0.90 [0.83, 0.99] | 0.89 [0.80, 0.98] |
| Omitting JPAD 2008 | 0.89 [0.84, 0.95] | 0.93 [0.84, 1.04] | 0.89 [0.82, 0.96] | 0.91 [0.84, 0.99] | 0.89 [0.81, 0.97] |
| Omitting JPPP 2014 | 0.90 [0.84, 0.95] | 0.94 [0.84, 1.04] | 0.90 [0.83, 0.98] | 0.91 [0.83, 0.99] | 0.89 [0.81, 0.98] |
| Pooled estimate RR | 0.90 [0.85, 0.95] | 0.95 [0.84, 1.06] | 0.89 [0.82, 0.96] | 0.91 [0.84, 0.99] | 0.89 [0.81, 0.97] |

**Supplemental Table 6 Results of leave-one-out method in sensitivity analysis for Hemorrhagic stroke, Major bleeding, ICH and GI bleeding outcomes**

|  | **Hemorrhagic stroke** | **Major Bleeding** | **ICH** | **GI bleeding** |
| --- | --- | --- | --- | --- |
| **Study** | **RR [95% CI]** | **RR [95% CI]** | **RR [95% CI]** | **RR [95% CI]** |
| Omitting HOT 1998 | 1.18 [0.94, 1.48] | 1.58 [1.28, 1.94] | 1.32 [1.11, 1.58] | 1.76 [1.32, 2.34] |
| Omitting TPT 1998 | 1.15 [0.92, 1.42] | 1.60 [1.32, 1.94] | 1.31 [1.10, 1.55] | 1.76 [1.36, 2.28] |
| Omitting PPP 2001 | 1.16 [0.93, 1.43] | 1.55 [1.30, 1.86] | 1.29 [1.09, 1.53] | 1.73 [1.34, 2.23] |
| Omitting WHS 2005 | 1.16 [0.90, 1.50] | 1.65 [1.34, 2.02] | 1.34 [1.11, 1.61] | 1.86 [1.40, 2.47] |
| Omitting POPADAD 2008 | 1.16 [0.93, 1.43] | 1.66 [1.39, 1.98] | 1.30 [1.09, 1.54] | 1.90 [1.49, 2.41] |
| Omitting AAA 2010 | 1.15 [0.93, 1.43] | 1.61 [1.33, 1.95] | 1.29 [1.08, 1.53] | 1.83 [1.40, 2.39] |
| Omitting ASCEND 2018 | 1.19 [0.95, 1.51] | 1.64 [1.34, 2.02] | 1.31 [1.09, 1.59] | 1.87 [1.40, 2.48] |
| Omitting ARRIVE 2018 | 1.19 [0.95, 1.48] | 1.58 [1.29, 1.94] | 1.32 [1.11, 1.58] | 1.75 [1.32, 2.33] |
| Omitting ASPREE 2018 | 1.12 [0.88, 1.43] | 1.60 [1.29, 1.99] | 1.21 [0.98, 1.49] | 1.82 [1.36, 2.45] |
| Omitting JPAD 2008 | 1.17 [0.94, 1.46] | 1.58 [1.31, 1.92] | 1.30 [1.09, 1.55] | 1.75 [1.35, 2.26] |
| Omitting JPPP 2014 | 1.08 [0.86, 1.36] | 1.46 [1.31, 1.63] | 1.26 [1.05, 1.51] | 1.59 [1.33, 1.89] |
| Pooled estimate RR | 1.16 [0.94, 1.44] | 1.58 [1.33, 1.88] | 1.31 [1.11, 1.56] | 1.76 [1.40, 2.22] |

**Supplemental Figure 1. Funnel plot with trim and fill method for major adverse cardiovascular events**

**Supplemental Figure 2. Funnel plot with trim and fill method for cardiovascular death.**

**Supplemental Figure 3. Funnel plot with trim and fill method for myocardial infarction event**

**Supplemental Figure 4. Funnel plot with trim and fill method for stroke event**

**Supplemental Figure 5. Funnel plot with trim and fill method for ischemic stroke**

**Supplemental Figure 6. Funnel plot with trim and fill method for hemorrhagic stroke**

**Supplemental Figure 7. Funnel plot with trim and fill method for major bleeding events**

**Supplemental Figure 8. Funnel plot with trim and fill method for intra-cranial hemorrhage**

**Supplemental Figure 9. Funnel plot with trim and fill method for gastrointestinal bleeding event**
